# CO_2_ oxidative coupling of methane using an earth-abundant CaO-based catalyst

**DOI:** 10.1038/s41598-019-51817-2

**Published:** 2019-10-29

**Authors:** Yongzheng Zhang, Yohei Cho, Akira Yamaguchi, Xiaobo Peng, Masahiro Miyauchi, Hideki Abe, Takeshi Fujita

**Affiliations:** 1 School of Environmental Science and Engineering, Kochi University of Technology, 185 Miyanokuchi, Tosayamada, Kami City, Kochi 782-8502 Japan; 2 Tokyo Institute of Technology, 2-12-1 Ookayama, Meguro-ku, Tokyo 152-8552 Japan; 3 National Institute for Materials Science, 1-1 Namiki, Tsukuba, Ibaraki 305-0044 Japan; 4 School of Physics and Physical Engineering, Qufu Normal University, Qufu, 273165 China

**Keywords:** Chemistry, Energy science and technology, Materials science, Nanoscience and technology

## Abstract

CO_2_ oxidative coupling of methane has been achieved by using CO_2_ as the oxidant. We explored various catalysts with the capability of producing C_2,3_ hydrocarbons and found that the use of a CaO-based oxide with sodium (Na) and chloride (Cl) allowed for remarkable direct methane conversion with a C_2,3_ yield of 6.6% at 950 °C. Microstructural characterisations showed that the optimal sample contained sodium carbonate (Na_2_CO_3_) covered with fine calcium oxide particles with chloride doping. Interestingly, sodium carbonate acted as a molten salt catalyst in this scenario. The synthesised active components are earth-abundant and can increase the possibility of achieving higher yields of hydrocarbons.

## Introduction

Effective utilisation of methane (CH_4_) and carbon dioxide (CO_2_) is important for realising a more sustainable society because these are the major components of natural and greenhouse gases. Catalytic reforming of CH_4_ with CO_2_ is known as dry reforming of methane (DRM) to syngas (H_2_ + CO): CH_4_ + CO_2_ → 2CO + 2 H_2_^1,2^. However, other possible reaction pathways to synthesise C_2_ or higher hydrocarbons are also possible, in which CO_2_ is used as an oxidant: 2CH_4_ + 2CO_2_ → C_2_H_4_ + 2CO + 2H_2_O and 2CH_4_ + CO_2_ →C_2_H_6_ + CO + H_2_O^3,4^. Although oxidative coupling of methane (OCM) using O_2_ as an oxidant has been known as a useful reaction to directly convert methane to C_2_ or higher hydrocarbons since the 1980s^5–8^, corresponding reactions using CO_2_ in CO_2_-OCM are quite challenging to achieve. So far, explorative studies investigating many oxides and their mixtures have been reported^3,9^, which attained C_2_ yields of 3–4% using modified CeO_2_ with CaO as the effective catalyst^10,11^, but the corresponding yields are still insufficient.

Based on previous knowledge and our explorative search for various catalysts, we found that mixed Ca- and Na-based oxides, synthesised via a chemical route with additive NaCl, show direct methane conversion with a remarkable C_2,3_ yield of ~6% at 950 °C. The polymerised complex (PC) method is the most studied and frequently used technique for the preparation of oxides because these methods can accurately control the final composition and yield pure and homogeneous oxides^12,13^. It was thus used in this study to ensure optimal results and reproducibility of experiments. Microstructural characterisation indicated that the product was uniform Na_2_CO_3_ covered with fine CaO particles with Cl doping. Interestingly, the Na_2_CO_3_ works as a molten salt catalyst at the reaction temperature of interest. The complex CaO-based oxides produced in this study are inexpensive and earth-abundant in the sea and would widen the opportunity to attain higher yields of hydrocarbons via CO_2_-OCM.

## Results and Discussion

**Figure 1:**
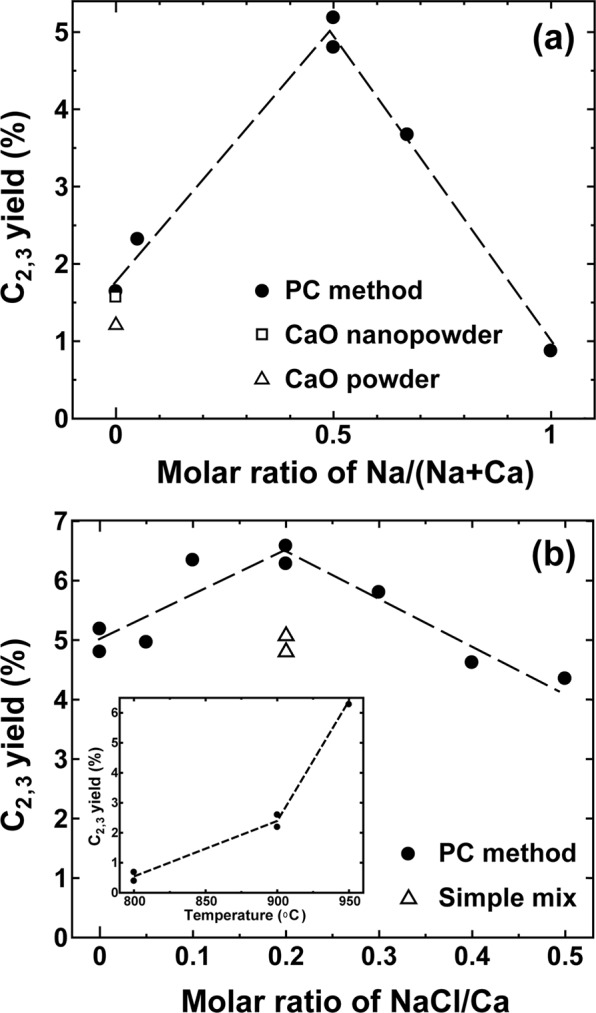
Catalytic performance at 950 °C. (**a**) C_2,3_ yield against the molar ratio of Na/Ca + Na in the chemical fabrication process. (**b**) C_2,3_ yield against the molar ratio of NaCl/Ca in the chemical fabrication process. The temperature dependence of the C_2,3_ yield is also shown in the inset of (**b**). Measurements were performed 20 min after reaching each temperature.

The first key finding was that the addition of Na to modified CaO oxides in the PC method was effective for hydrocarbon generation. Figure 1(a) shows the dependence of the C_2,3_ yield on the molar ratio of Na to Ca used. When the ratio was 0.5, i.e., equal moles of Ca and Na were used, the highest C_2,3_ yield of ~5% was obtained. For comparison, the corresponding data for commercial CaO nanopowder (Sigma-Aldrich, 98%, Product ID: 634182) with a large surface area and conventional CaO powder (Wako, 99.9%, 036-13572) are also shown, and the results indicate that the use of only CaO cannot give the high C_2,3_ yield.

The Cl element is a well-known promotor of CH_3_ radicals^14^. Therefore, in order to attain higher C_2,3_ yields, we tested various chlorides as additives under the conditions that gave the highest C_2,3_ yield in Fig. 1(a). As the second key finding, we found that the addition of NaCl was also effective in improving the yield. Figure 1(b) shows the dependence of the yield on the molar ratio of NaCl to Ca: a 20% molar ratio of NaCl to Ca gave a ~6% yield. In the ternary phase diagram for the CaCO_3_–Na_2_CO_3_–NaCl system^15,16^, the addition of NaCl lowers the melting temperature (~690 °C) effectively around the optimal composition and successful doping of Cl, and more homogeneous dispersions of composites may be realised in the synthesis process close to this composition. We summarise the representative results for the catalytic performance in Table 1. The temperature dependence of the C_2,3_ yield is also shown in the inset of Fig. 1(b), according to which 950 °C was the optimum reaction temperature, which was instrumentally the upper limit in our experimental setup. For comparison, we also synthesised the catalysts at the optimal composition via a simple mixing procedure, but the performance (~5%) was lower than that attained by the PC method. Therefore, we considered mixing by the PC method as the optimal technique and further characterised the microstructure of the optimised sample. Notably, the product without Ca obtained by the PC method shows poor performance (0.8~1%).

**Table 1:** Representative performance of the optimal sample and commercial CaO nanopowder.

Catalyst	CH_4_ conversion (%)	C_2,3_ yield (%)	Selectivity (%)				
			C_2_H_2_	C_2_H_4_	C_2_H_6_	C_3_H_6_	C_3_H_8_
Ca:Na:NaCl (1:1:0.2)^a^ at 950 °C	18.9	6.6	9.2	63.3	21.5	3.4	2.6
Ca:Na:NaCl (1:1:0.2)^a^ at 900 °C	23.1	2.5	0.9	42.6	55.6	0.9	0
CaO nanopowder at 950 °C	9.3	1.6	4.7	64	31.3	0	0

Measurements were carried out 20 min after reaching each temperature.

^a^Optimal molar ratio of the mixture in the polymerised complex method.

**Figure 2:**
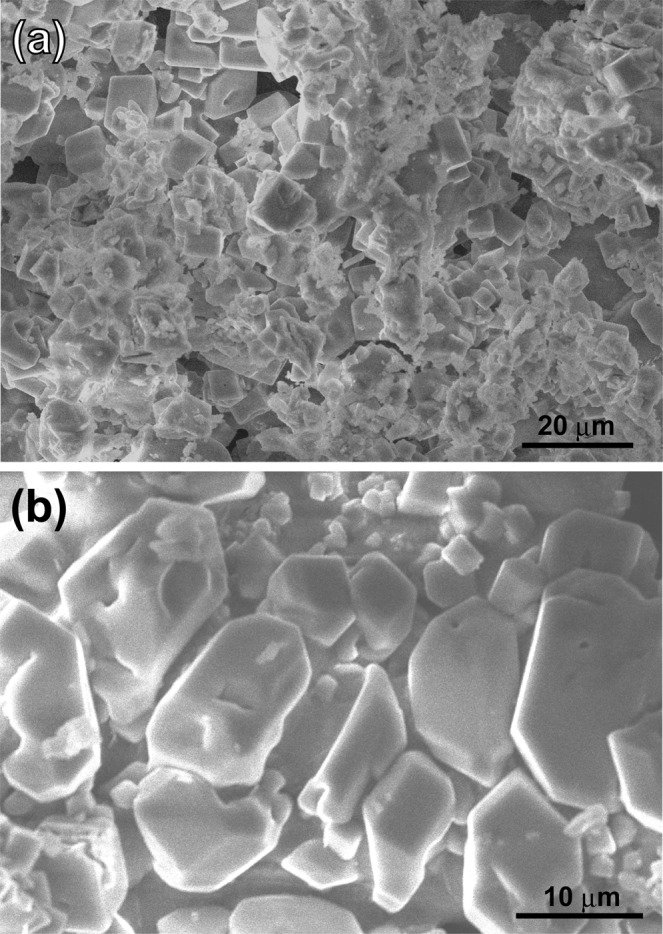
(**a**,**b**) SEM images for the optimal sample at low and high magnifications.

**Figure 3:**
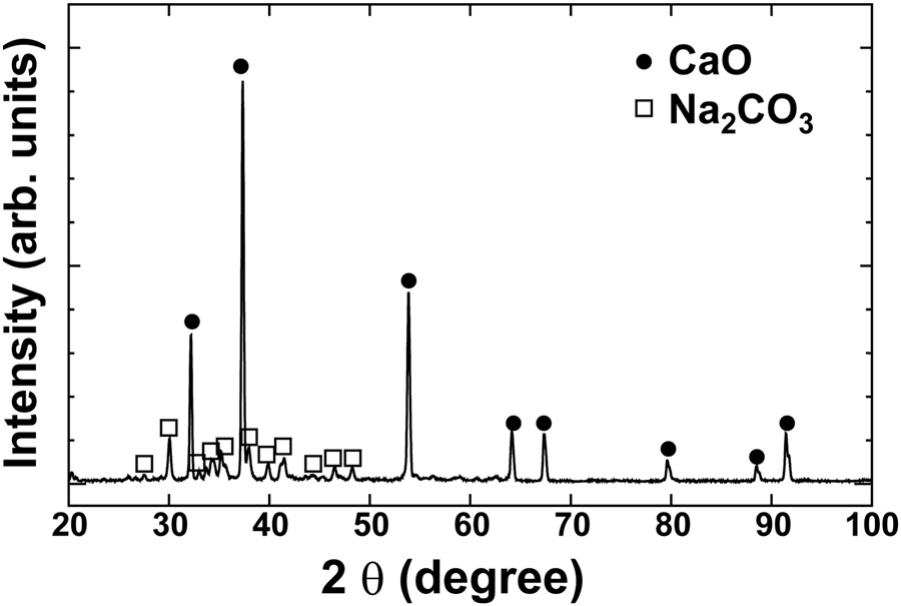
X-ray diffraction profile for the optimal sample, showing two phases of CaO (JCPDF#37-1497) and Na_2_CO_3_ (#37-0451).

**Figure 4:**
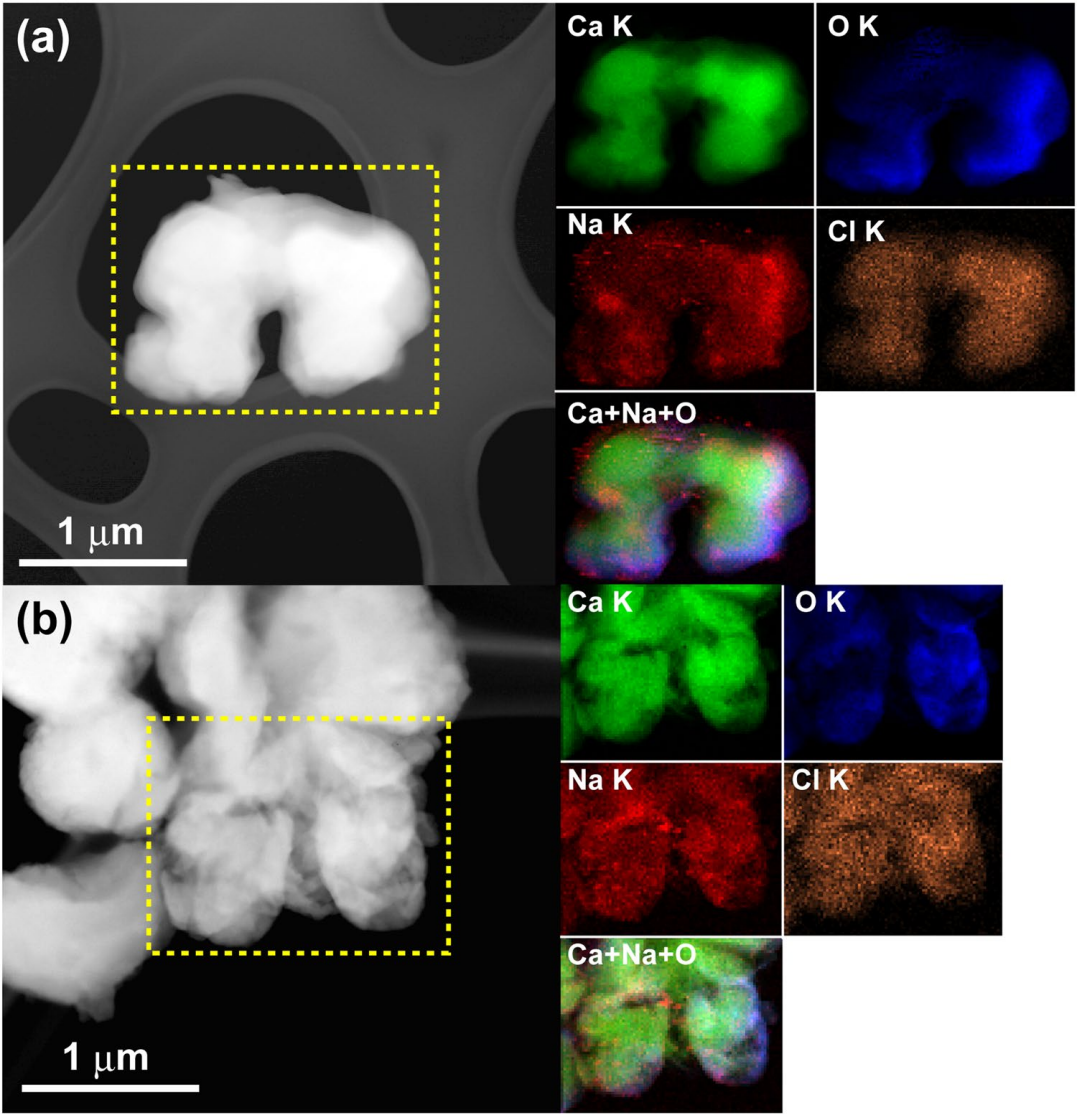
(**a**,**b**) STEM image and EDS chemical maps of the selected area, showing the distributions of Ca (green), Na (red), O (blue), and Cl (orange) and the mixture of Ca + Na + O.

Figure 2 shows the SEM images at low and high magnifications. The particle sizes varied from 10 to 20 microns, and the nominal composition was Ca_13.7_Na_13.3_C_8.4_O_64.4_Cl_0.2_ (at.%) as determined by SEM-EDS analysis. EDS analysis results for the samples with different NaCl/Ca ratios are summarised in Table S1. The doping of Cl was also confirmed, and the Brunauer–Emmett–Teller (BET) surface area was calculated to be 4.6 m^2^/g (Fig. S1). Unexpectedly, the X-ray diffraction profile shows that the optimal sample contains two phases, CaO (JCPDF#37-1497) and Na_2_CO_3_ (#37-0451), as shown in Fig. 3. We also recorded the X-ray diffraction profiles (XRD) patterns for samples with different NaCl/Ca ratios (Fig. S2 in Supporting Information). The XRD patterns indicated that all the samples contain two phases, CaO and Na_2_CO_3_. Moreover, the energy-dispersive X-ray spectroscopy (EDS) mapping analysis and scanning transmission electron microscopy (STEM) data, as shown in Fig. 4, clearly displays the uniform coverage by Na of Na_2_CO_3_ over CaO fine particles. The melting temperature of Na_2_CO_3_ was 851 °C, and the optimal temperature (950 °C) is far above that. Therefore, Na_2_CO_3_ works as a molten salt catalyst, and the melting behaviour observed was very stark from the morphology change whenever we checked the sample after the catalytic tests, as shown in Fig. S3. The TGA results also confirmed the melting state of Na_2_CO_3_ at 950 °C, as shown in Fig. S4.

**Figure 5:**
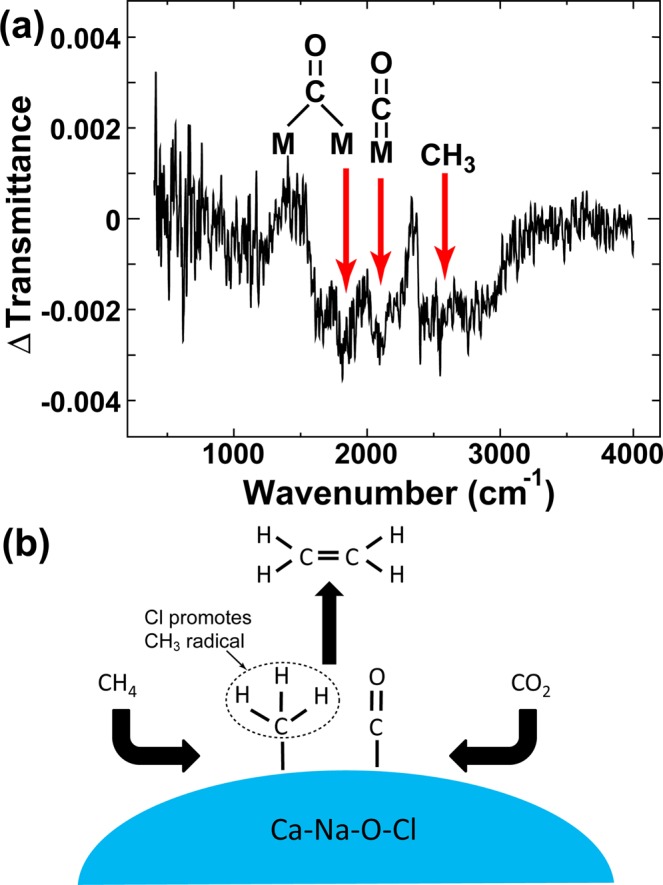
(**a**) FTIR difference spectrum for the optimal sample, which was obtained by subtracting the spectrum obtained for 25 min after leaching at 900 °C from that obtained for 30 min. (**b**) Proposed mechanism for the formation of hydrocarbons over the Ca–Na–O–Cl catalyst.

We performed *in situ* Fourier transform infrared (FTIR) experiments to clarify the mechanism of hydrocarbon synthesis, at the reaction temperature shown in Fig. 5(a), and we confirmed the existence of carbonyl (M=C=O) and methane radical groups (-CH_3_). The suggestive mechanism has been outlined in Fig. 5(b). Because Na_2_CO_3_ can decompose to Na_2_O + CO_2_ above the melting temperature^17^, Na_2_O on the surface could convert CH_4_ to CH_3_ radicals with CaO^18,19^ with the assistance of Cl dopants efficiently. Accordingly, two or more CH_3_ radicals were coupled towards the production of C_2,3_ hydrocarbons, as has been reported previously^11^. Further investigations are needed to provide a clearer mechanism.

**Figure 6:**
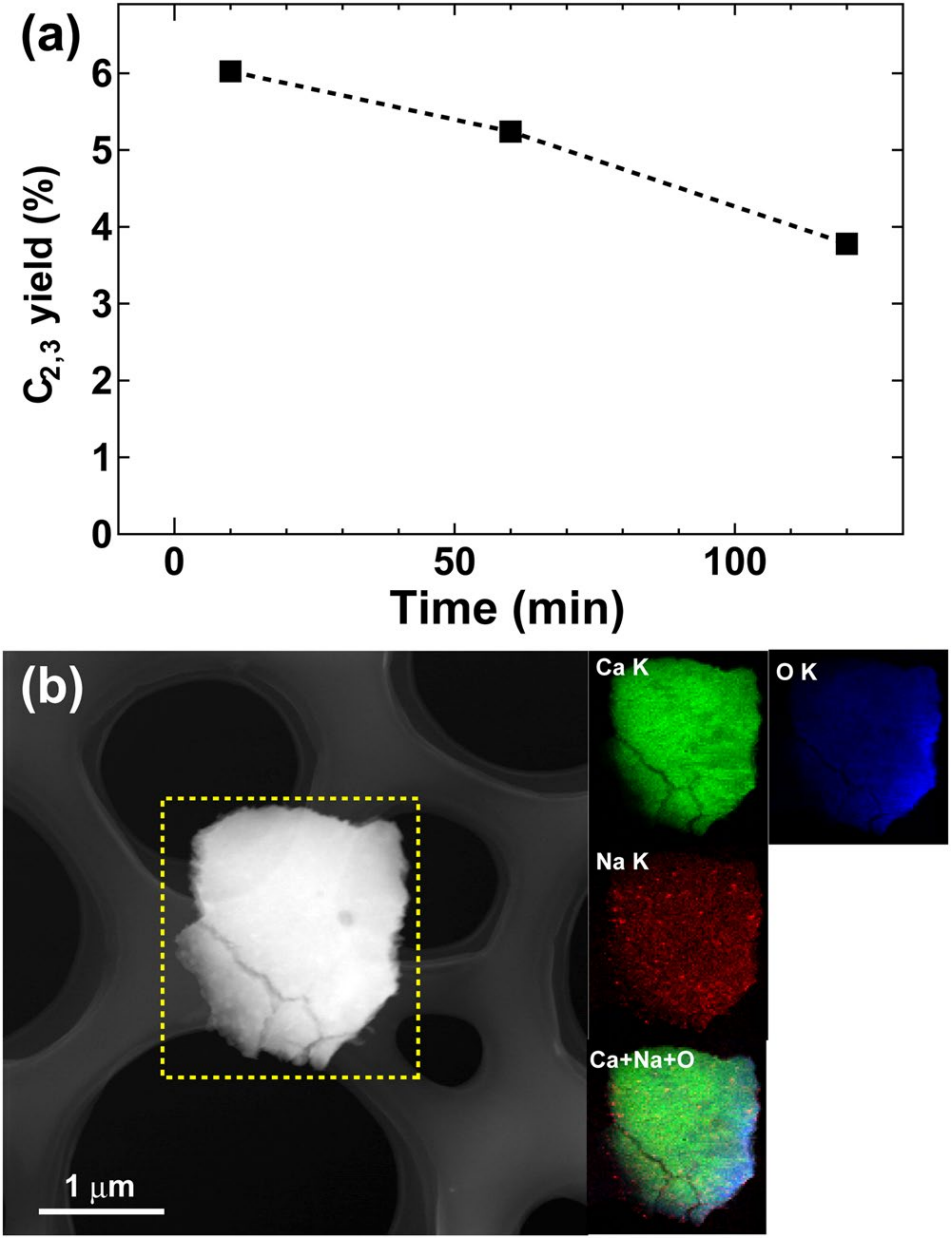
(**a**) Durability test for the optimal sample. (**b**) STEM image and EDS chemical maps of the spent sample, showing the distributions of Ca (green), Na (red), and O (blue) and the mixture of Ca + Na + O. Cl elements were not detected sufficiently for visualisation.

We checked the durability of the optimal catalyst, as shown in Fig. 6(a) and the C_2,3_ yield gradually decreased over time. STEM imaging with EDS mapping after the catalytic test was employed as shown in Fig. 6(b) and it showed a reduction in the Na coverage and reduced detection of Cl over CaO particles, thus demonstrating the major reason for degradation of hydrocarbon production. The XRD patterns and X-ray photoelectron spectroscopy (XPS) profiles after the experiment also showed the signal reduction of Na and Cl (Figs S5–S7). Regarding the XPS profiles, by comparison with the Ca profile in the sample with (Fig. S6a) and without 20% NaCl (Fig. S6c), two kinds of peaks of Ca 2p_3/2_ can be observed as shown in Fig. S6c. Their binding energies are about 345.0 eV and 346.5 eV, which correspond to Ca metallic bonding and CaO insulating ones, respectively^20^. Moreover, the spitting of Cl 2p_3/2_ as shown in Fig. S6b demonstrates that Ca-Cl and Na-Cl are in the sample with NaCl. These results indicate that NaCl in the CaO-Na_2_CO_3_ could contribute to the refusion of CaO and Na_2_CO_3_. After long-term testing of our optimal sample, a new peak located at 347.0 eV appeared as shown in Fig. S7c, which corresponds to CaCO_3_. It indicates that some CaO turned to CaCO_3_ after long-term testing. It should be noted that the content of Cl goes to zero after long-term tests.

Since mass reduction via evaporation is a major concern of alkali molten catalysts in general^21^, this issue must be given attention to further improve our results. Suppressing mass reduction is thus a topic of future research underway in our group as well as the design of other suitable reactor designs such as a bubbling system^22^ and/or increasing the pressure of the gas atmosphere to gain more hydrocarbons.

## Conclusion

In summary, we have fabricated a complex CaO-based oxide by the PC method, in which we achieved uniform Na_2_CO_3_ covered with CaO fine particles with Cl dopants. The optimal composition gave the highest C_2,3_ yield of 6.6% at 950 °C via CO_2_-OCM. Na_2_CO_3_ works as a molten salt catalyst at the reaction temperature, and the suppression of mass reduction is an issue requiring further studies for improvement. Because the proposed catalysts are inexpensive and truly earth abundant, our method could contribute to achieving higher yields of hydrocarbons in the future.

## Methods

### Sample preparation

The following procedure is based on a modified polymerised complex (PC) method^23^ to achieve the optimum composition in this study. The molar ratio of each chemical can be varied, as described in the results. Calcium citrate (Ca_3_(C_6_H_5_O_7_)_2_•4H_2_O, Wako, 97.0%), trisodium citrate (C_6_H_5_Na_3_O_7_, Wako, 99.0%), and L-aspartic acid (C_4_H_7_NO_4_, Wako, 99.0%) were dissolved in deionised water at a molar ratio of 1:1:9. After ultrasonic treatment for 5 min, 69% (wt.%) nitric acid (HNO_3_, Wako) was added dropwise until all of the calcium citrate powder in the solution dissolved. Next, sodium chloride (NaCl, Wako, 99.5%) was dissolved in a solution with a 60% molar content of calcium citrate, i.e., NaCl:Ca = 0.2:1 (by mol). After drying at 120 °C for 12 h in the oven, a pale-yellow precursor was formed. A final annealing process was conducted in an alumina combustion boat at 700 °C for 2 h in air. After the heat treatment, a white powder was obtained after cooling naturally to room temperature.

For comparison with the PC method, the following simple mixing procedure for obtaining the optimal composition was employed: 0.1 mol calcium oxide (CaO, Wako, 99.9%) was dispersed into 100 mL of deionised water and then 0.1 mol sodium hydroxide (NaOH, Wako, 97.0%) and 0.02 mol sodium chloride (NaCl, Wako, 99.5%) were added to the solution. After ultrasonic treatment for 5 min, the turbid solution was dried at 120 °C for 12 h in the oven. The precursor was transferred to an alumina combustion boat and a final annealing process was conducted at 700 °C for 2 h in air. After the heat treatment, white powders were obtained.

### Characterisation

Microstructures of the obtained catalysts were characterised by transmission electron microscopy (TEM, JEM-2100F, JEOL, equipped with aberration correctors, for the image- and probe-forming lens systems, CEOS GmbH) and energy-dispersive X-ray spectrometry (EDS, JED-2300T, JEOL). High-resolution TEM (HRTEM) and scanning TEM (STEM) observations were conducted at an accelerating voltage of 200 kV with the Cs correctors. The samples were transferred onto a Cu grid without the use of a uniform carbon support film^24^. The microstructures of the samples were also characterised by using a field-emission scanning electron microscopy (SEM; JEOL JIB-4600F, 15 kV) system equipped with an X-ray energy-dispersive spectrometry apparatus. X-ray diffraction profiles (XRD) were obtained by using a Rigaku SmartLab X-ray diffractometer with Cu Kα radiation at 30 kV^24^. The samples were analysed by thermogravimetry-differential thermal analysis (TG-DTA, NETZCH, STA 2500) under nitrogen. The temperature was increased at the rate of 20 K/min. The chemical state of the samples was measured by X-ray photoelectron spectroscopy (XPS; AXIS ultra DLD, Shimadzu) with a monochromated AlKα radiation source.

### Catalytic experiments

The desired sample (100 mg) was loaded into a 4-mm-diameter quartz tube and tested by using a continuous-flow fixed-bed microreactor under atmospheric pressure. The quantities of CH_4_, CO, H_2_, CO_2_, C_2_H_2_, C_2_H_4_, C_2_H_6_, C_3_H_6_, and C_3_H_8_ were monitored and evaluated by using an on-line gas analyser (BELMass, MicrotracBEL) and a gas chromatograph (GC-2014 and Trasera, Shimadzu, Japan) equipped with a thermal conductivity detector, flame ionisation detector, and barrier ionisation detector^24^. The reactant gas containing 1 vol.% CH_4_, 1 vol.% CO_2_, and He to compensate was introduced into the reactor at a space velocity (SV) of 10 cm^3^ min^−1^ (W/F = 0.6 g s cm^−3^). The optimum reaction temperature to obtain the maximum C_2,3_ yield was 950 °C and was instrumentally the upper limit. The calculations were conducted by using the following formulae^25^:

$${{\rm{CH}}}_{4}\,\mathrm{conversion}\,[ \% ]=\frac{{[{{\rm{CH}}}_{{\rm{4}}}]}_{{\rm{in}}}-{[{{\rm{CH}}}_{{\rm{4}}}]}_{{\rm{out}}}}{{[{{\rm{CH}}}_{{\rm{4}}}]}_{{\rm{in}}}}\times 100$$

$${{\rm{C}}}_{2,3}{\rm{yield}}\,({\rm{carbon}}\,{\rm{base}})[ \% ]=\frac{2{[{{\rm{C}}}_{2}{{\rm{H}}}_{{\rm{2}}}]}_{{\rm{out}}}+2{[{{\rm{C}}}_{2}{{\rm{H}}}_{4}]}_{{\rm{out}}}+2{[{{\rm{C}}}_{2}{{\rm{H}}}_{6}]}_{{\rm{out}}}+{3[{\rm{C}}}_{{\rm{3}}}{{\rm{H}}}_{{\rm{6}}}{]}_{{\rm{out}}}+{3[{\rm{C}}}_{{\rm{3}}}{{\rm{H}}}_{{\rm{8}}}{]}_{{\rm{out}}}}{{[{{\rm{CH}}}_{{\rm{4}}}]}_{{\rm{in}}}}\times 100$$

Therefore, selectivity of ethane, as an example (carbon base) [%]

$$=\,\frac{2{[{{\rm{C}}}_{2}{{\rm{H}}}_{{\rm{6}}}]}_{{\rm{out}}}}{2{[{{\rm{C}}}_{2}{{\rm{H}}}_{{\rm{2}}}]}_{{\rm{out}}}+2{[{{\rm{C}}}_{2}{{\rm{H}}}_{4}]}_{{\rm{out}}}+2{[{{\rm{C}}}_{2}{{\rm{H}}}_{6}]}_{{\rm{out}}}{+3[{\rm{C}}}_{{\rm{3}}}{{\rm{H}}}_{{\rm{6}}}{]}_{{\rm{out}}}{+3[{\rm{C}}}_{{\rm{3}}}{{\rm{H}}}_{{\rm{8}}}{]}_{{\rm{out}}}}\times 100$$

where […]_in_ and […]_out_ represent the gas concentrations in the feed gas and effluent gas, respectively.

### Surface area measurements and analysis

The Brunauer–Emmett–Teller (BET) surface areas of the samples were measured at 77 K by using a BELSORP-MAX II (MicrotracBEL Japan, Inc.). Each sample was heated at 120 °C under vacuum for 24 h prior to measurement, and the mass of each sample was measured by using a balance^24^.

FTIR spectra of the catalyst surfaces were measured at the operating temperature by using a JASCO 6100 FTIR system equipped with a heat chamber (ST-Japan). Each sample (5 mg) was loaded onto the sample stage, and the reactant gas containing 1 vol.% CH_4_, 1 vol.% CO_2_, and Ar to compensate was introduced into the environmental cell at a rate of 10 cm^3^ min^−1^^24^.

## Supplementary information

Supplementary Material

## Data Availability

The datasets generated during and/or analysed during the current study are available from the corresponding author on reasonable request.
