# Supplementary material for: CO2 oxidative coupling of methane using an earth-abundant CaO-based catalyst
Source: Sci Rep. 2019 Oct 29;9:15454. doi: 10.1038/s41598-019-51817-2 (PMC6820859; doi:10.1038/s41598-019-51817-2)
Supplement: Supplementary file 1 — Supplementary Material [file 41598_2019_51817_MOESM1_ESM.pdf]

Supporting Information

CO_2 oxidative coupling of methane using an earth-abundant CaO-based catalyst

Yongzheng Zhang, Yohei Cho, Akira Yamaguchi, Xiaobo Peng, Masahiro Miyauchi,

Hideki Abe, Takeshi Fujita

Email: fujita.takeshi@kochi-tech.ac.jp

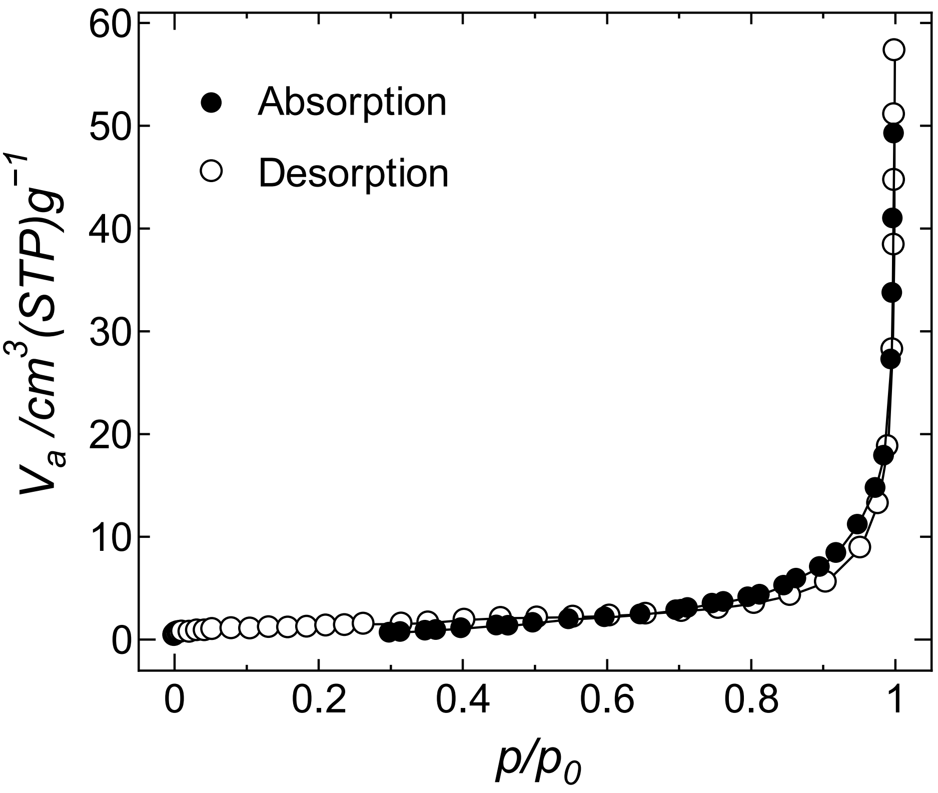

Figure S1. Nitrogen absorption and desorption measurements of the optimal product in this study. The BET surface area is 4.6 m^2/g.

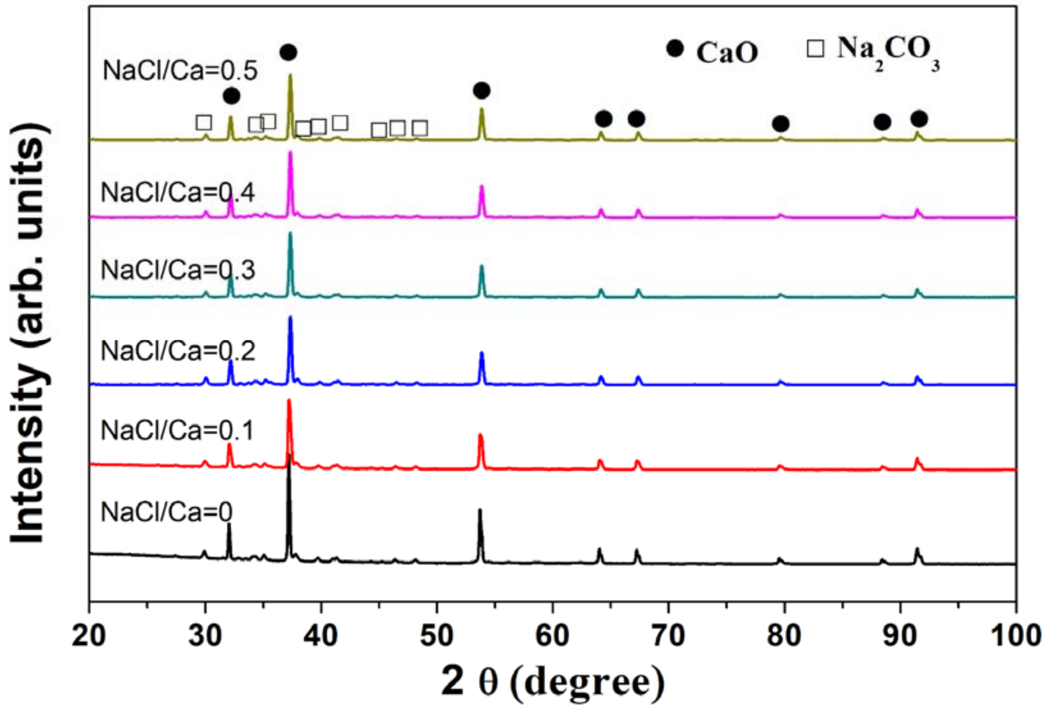

Figure S2. X-ray diffraction profiles for different ratios of NaCl/Ca samples. The XRD patterns indicate that all of the optimal samples contain two phases, CaO (JCPDF#37-1497) and Na_2CO_3 (#37-0451).

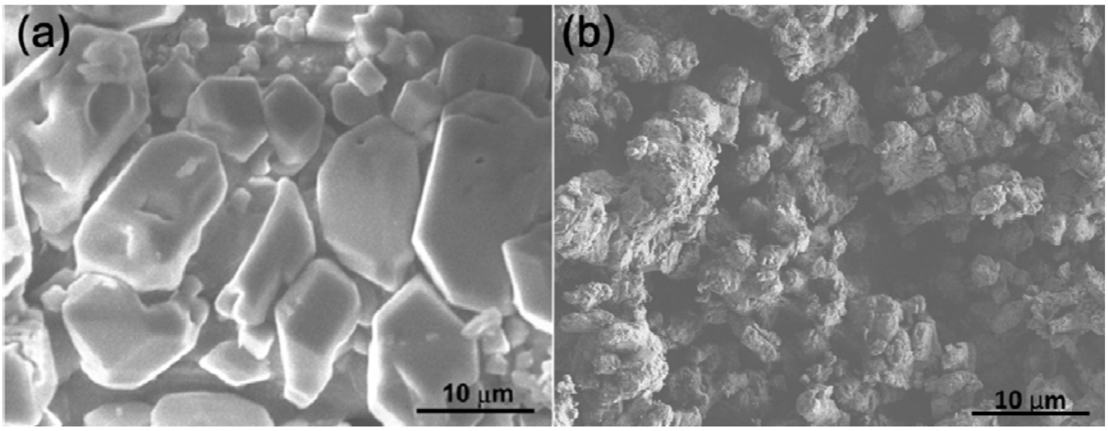

Figure. S3. SEM images of the optimal sample before (a) and after (b) the DRM test.

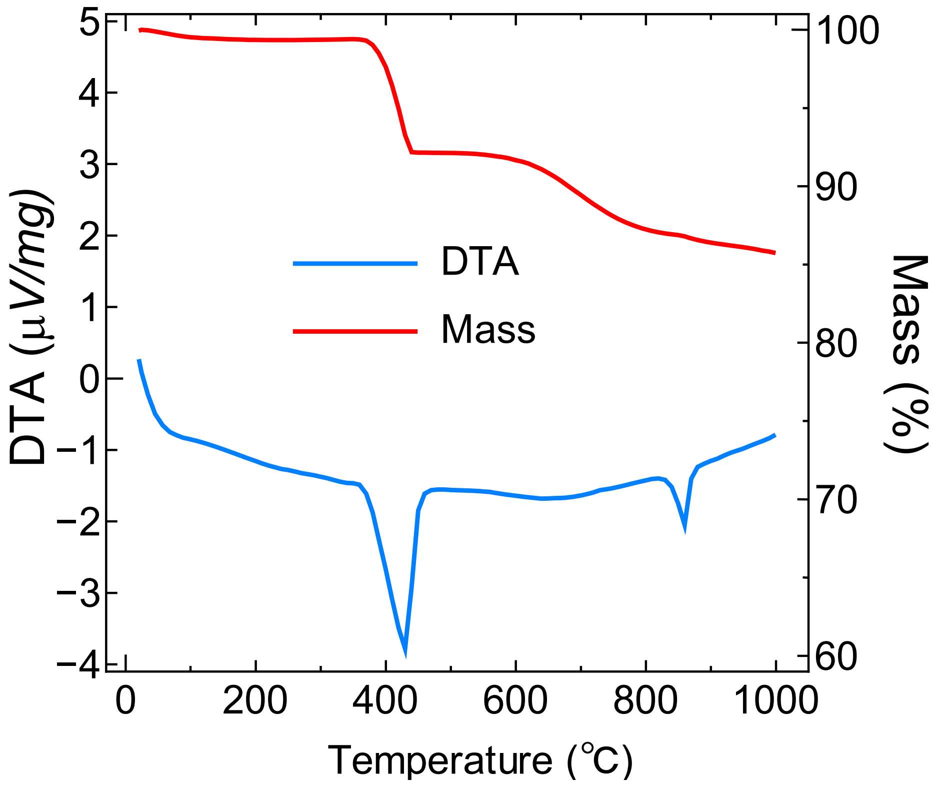

Figure S4. Thermogravimetry (TG) and differential temperature analysis (TDA) results of the optimal product at elevated temperatures up to 1000 °C. The drop in the DTA curve at 430 °C corresponds to water evaporation from residual $Ca(OH)_2$, $Ca(OH)_2 \rightarrow CaO + H_2O$. The drop in the DTA curve at 860 °C corresponds to melting of Na_2CO_3, where the nominal melting temperature is 851 °C.

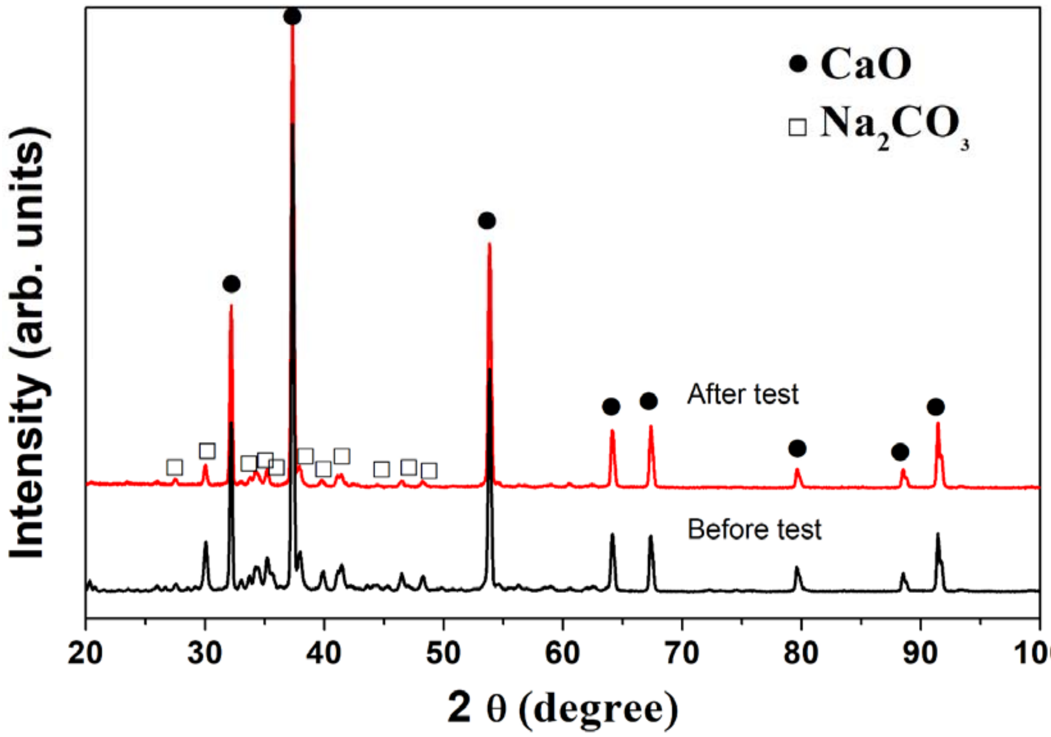

Figure S5. X-ray diffraction profile for the optimal sample after the catalytic test, showing the reduced signals of Na_2CO_3 because of evaporation. The main phases of both samples are CaO (JCPDF#37-1497) and Na_2CO_3 (#37-0451). However, the intensity of the Na_2CO_3 phase becomes weaker after the test, thus indicating the molten phase of Na_2CO_3 and evaporation during the test.

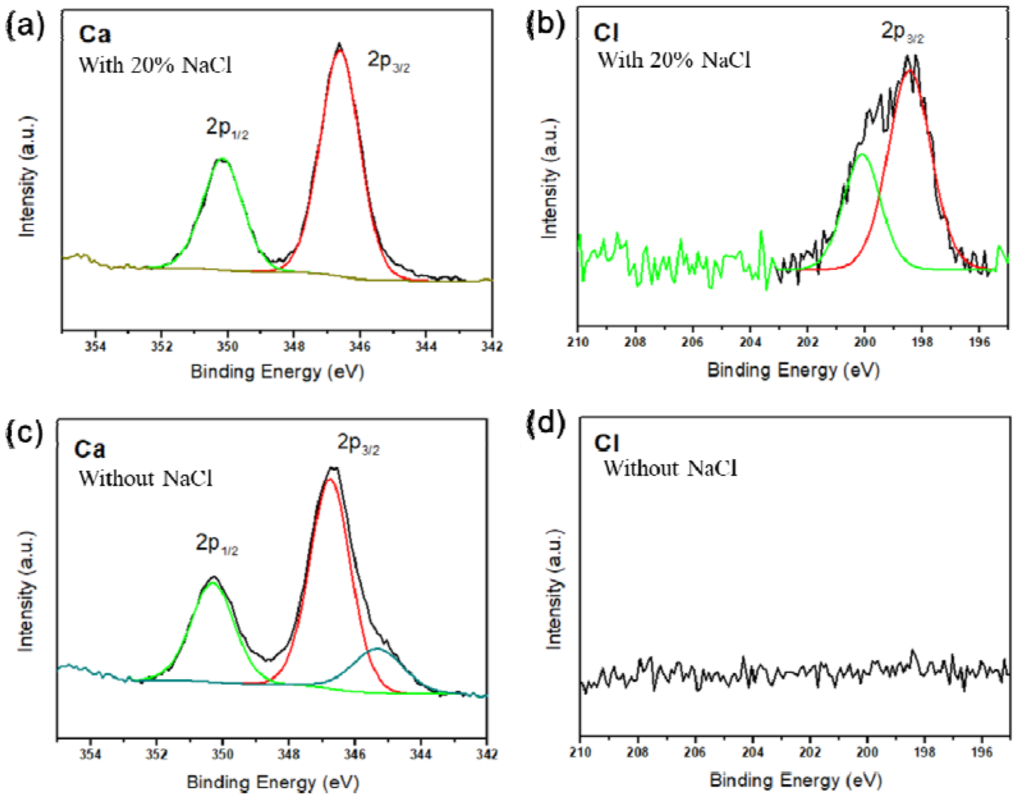

Figure S6. XPS profiles of Ca and Cl in the sample with 20% NaCl (a, b) and without NaCl (c, d).

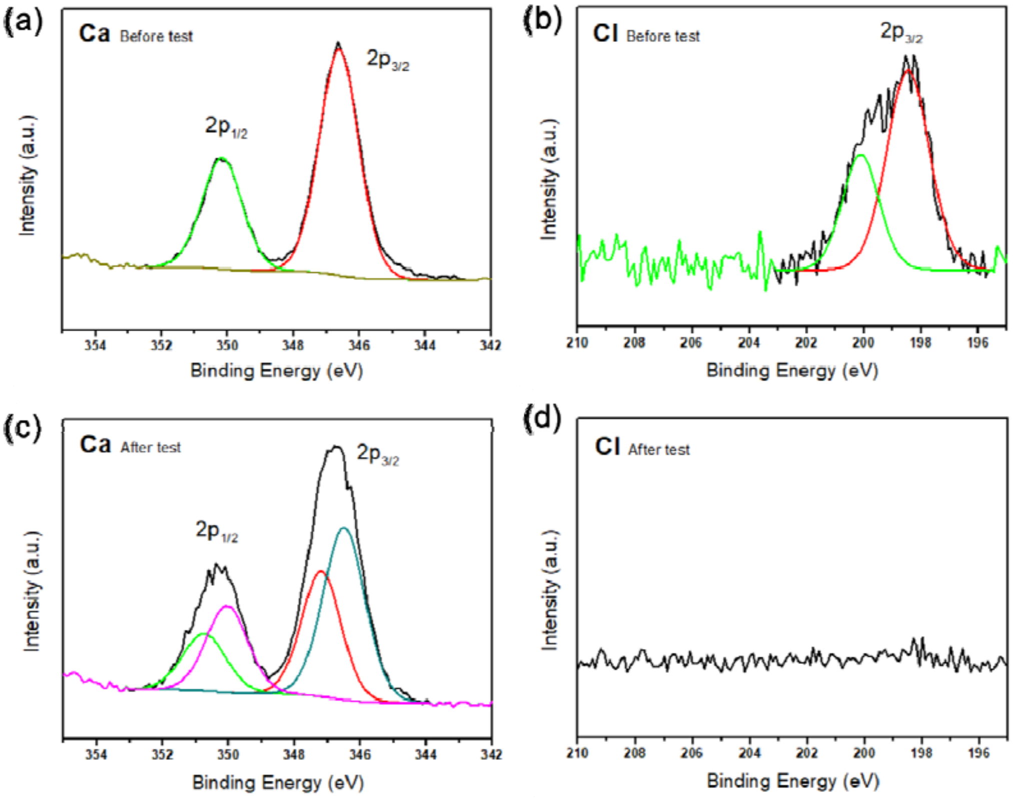

Figure S7. XPS profiles of Ca and Cl in the optimal sample. (a, b) before and (c, d) after the long-term testing at 950 °C for 10 h.

Table S1. EDS analysis of samples with different NaCl/Ca ratios

Sample (NaCl/Ca)	Contents (at.%)				
	Ca	Na	C	O	Cl
0	12.13	11.64	7.21	69.02	0
0.1	12.51	12.01	8.32	67.10	0.06
0.2	13.65	13.34	8.38	64.42	0.21
0.3	13.21	11.93	8.76	65.82	0.28
0.4	12.55	12.27	8.54	66.21	0.43
0.5	12.47	11.78	7.43	67.86	0.46
